# Validation studies of verbal autopsy methods: a systematic review

**DOI:** 10.1186/s12889-022-14628-1

**Published:** 2022-11-29

**Authors:** Buddhika P. K. Mahesh, John D. Hart, Ajay Acharya, Hafizur Rahman Chowdhury, Rohina Joshi, Tim Adair, Riley H. Hazard

**Affiliations:** 1 Melbourne School of Population and Global Health, The University of Melbourne, Carlton, Victoria, Australia; 2 The George Institute for Global Health, New Delhi, India; 3 School of Population Health, University of New South Wales, Sydney, Australia

**Keywords:** Verbal autopsy, Verbal autopsy mode, Validation, Cause of death, Civil registration and vital statistics

## Abstract

**Background:**

Verbal autopsy (VA) has emerged as an increasingly popular technique to assign cause of death in parts of the world where the majority of deaths occur without proper medical certification. The purpose of this study was to examine the key characteristics of studies that have attempted to validate VA cause of death against an established cause of death.

**Methods:**

A systematic review was conducted by searching the MEDLINE, EMBASE, Cochrane-library, and Scopus electronic databases. Included studies contained 1) a VA component, 2) a validation component, and 3) original analysis or re-analysis. Characteristics of VA studies were extracted. A total of 527 studies were assessed, and 481 studies screened to give 66 studies selected for data extraction.

**Results:**

Sixty-six studies were included from multiple countries. Ten studies used an existing database. Sixteen studies used the World Health Organization VA questionnaire and 5 studies used the Population Health Metrics Research Consortium VA questionnaire. Physician certification was used in 36 studies and computer coded methods were used in 14 studies. Thirty-seven studies used high level comparator data with detailed laboratory investigations.

**Conclusion:**

Most studies found VA to be an effective cause of death assignment method and compared VA cause of death to a high-quality established cause of death. Nonetheless, there were inconsistencies in the methodologies of the validation studies, and many used poor quality comparison cause of death data. Future VA validation studies should adhere to consistent methodological criteria so that policymakers can easily interpret the findings to select the most appropriate VA method.

**Prospero Registration:**

CRD42020186886.

**Supplementary Information:**

The online version contains supplementary material available at 10.1186/s12889-022-14628-1.

## Background

Accurate and reliable mortality data are crucial for making informed decisions about public health policy and interventions [1]. However, globally only one in four countries have well-functioning death registration systems and only one in three deaths are assigned a specific cause with policy utility [2–4]. In resource limited settings, where the majority of deaths occur out-of-hospital, traditional autopsy or cause of death certification by medical doctors is not practical for cause of death determination [5]. Verbal autopsy (VA) has emerged as a solution for collecting information on causes of death in low-income countries [1].

VA involves a trained interviewer administering a structured questionnaire to a carefully selected respondent who was with the deceased during the final illness [1]. Historically, physicians evaluated VA responses to assign a cause of death, but more recently there have been several computer-driven automated algorithms that assign cause of death based on VA [6–8]. The relative performance of these different diagnostic algorithms has also been evaluated yet remains controversial [9].

VA has been used increasingly beyond research projects by countries’ routine civil registration and vital statistics systems over the past 10 years [1]. The quality of cause of death data from VA will therefore directly impact health policy in many settings. Despite this, to our knowledge there has been no attempt to systematically review the quality of studies validating VA methods.

This review aims to describe the main characteristics of VA validation studies, including the VA questionnaire used, comparator dataset, and metrics to evaluate agreement. This review is a critical knowledge synthesis and discussion of the strengths and weaknesses of the VA validation processes. The findings of the review may facilitate the establishment of guidelines for VA validation processes.

## Methods

This study was registered with PROSPERO (registration number: CRD42020186886) [10]. The review process and reporting were done according to the Preferred Reporting Items for Systematic Reviews and Meta-Analyses (PRISMA) guidelines [11].

### Search strategy

A search strategy was developed and refined with librarian support (Additional File 1). MEDLINE, EMBASE, Cochrane-library and Scopus electronic databases were searched from the inception up to the June 2020. The search resulted in 1008 articles (i.e. 294, 307, 69, 338 articles respectively from each database). Those citations were imported into the EndNote-X9 reference manager application for deduplication. Following deduplication, there were 481 articles. A secondary search was conducted from July 2020 to January 2022 to identify new publications. An additional 35 studies were screened, and two were eligible for inclusion. Two reviewers (BPKM, HRC) conducted the search in parallel and independently.

### Eligibility criteria and study selection

Three screening questions were used in the study selection: 1) Does the study/article include a VA component (i.e. interviews with relevant respondents to determine cause of death); 2) Does the study include a validation component (i.e. comparison of cause of death assigned by the VA with another source that is not solely based on the data collected in the VA; and 3) Does the article include original analysis or a reanalysis (i.e. not a protocol or a comment/correspondence relating to another study).

**Fig. 1:**
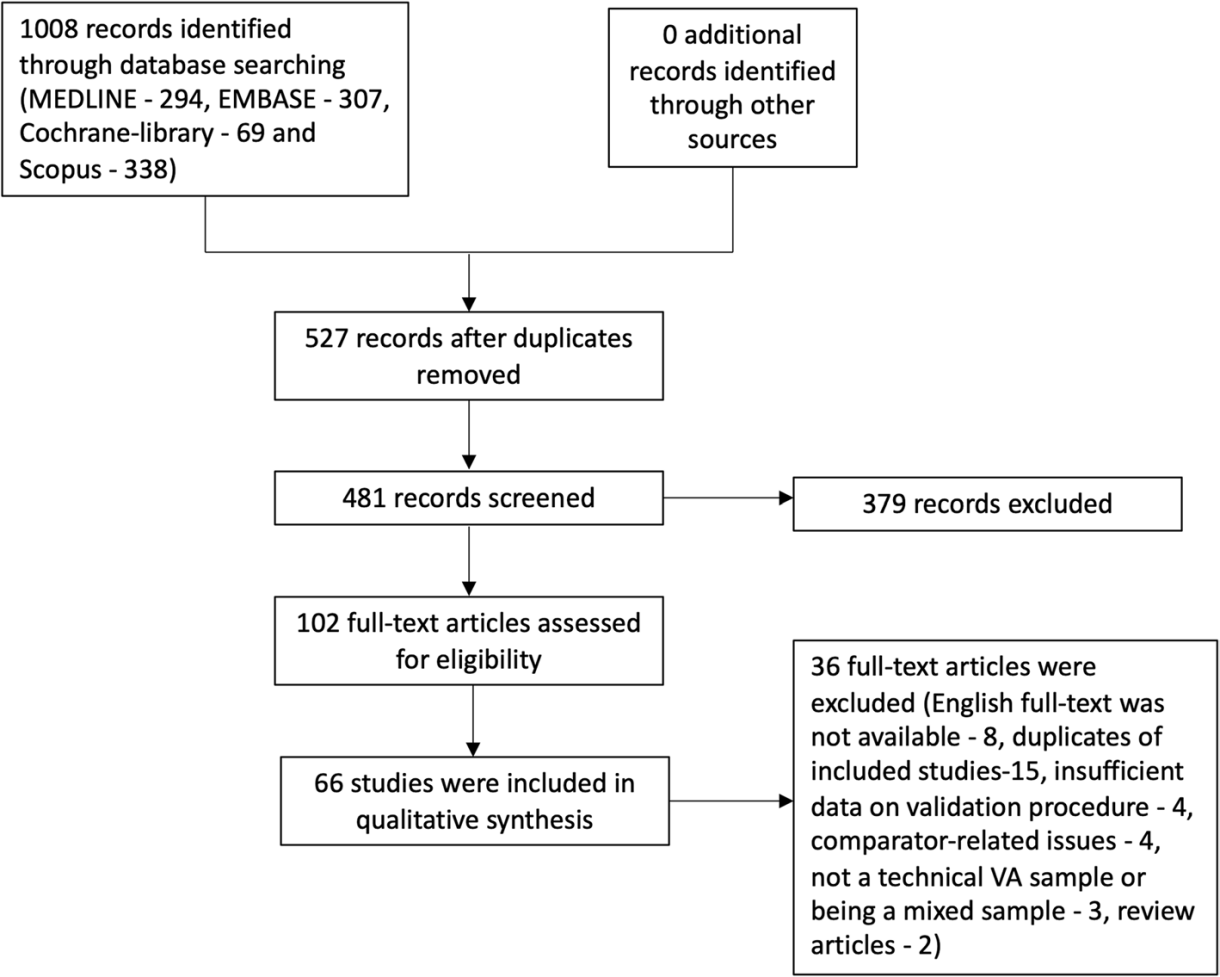
Flow diagram of study selection

In the first round, titles and abstracts were reviewed by two authors (BPKM, HRC). In the second round, full texts were reviewed. Two authors (BPKM, HRC) independently selected studies with the DistillerSR application. Discrepancies were reviewed by a third author (RJ). At the end of the second round, 66 articles were selected for data extraction by two authors (BPKM and JDH). The flow diagram of study selection is shown in Fig. 1.

### Risk of bias assessment

Risk of bias assessment was done with a modified tool comprised of 10 domains [12, 13]. Two authors (BPKM, AA) assessed the risks independently and any discrepancy was attended by a third (RHH).

- Domain 1: Study’s target population/dataset involved was a close representation of the population of a defined geographical area/s (national or sub-national) OR a close representation of the population presenting to a healthcare institution/s in relation to socio-demographic characteristics.
- Domain 2: Random selection was used to select the sample OR the total target population/dataset was covered.
- Domain 3: Non-response bias was minimal.
- Domain 4: Appropriate descriptive statistics have been used in describing the comparison.
- Domain 5: Appropriate summary statistics have been used for the comparison.
- Domain 6: Comparator had been clearly described.
- Domain 7: A validated instrument (e.g. questionnaire) was used for data collection.
- Domain 8: Observers were blinded to previous findings.
- Domain 9: Comparator had been determined before the data collection.
- Domain 10: Clear descriptions have been given on the competence of those who were involved in data collection and analysis.

All articles were assessed for each domain and one of the three responses was recorded for each: low-risk, unclear, and high-risk. A risk of bias assessment was summarized using a colour code system.

### Data extraction and narrative synthesis

Seventeen variables were extracted from the selected studies: 1) setting; 2) composition/details of the VA tool used; 3) relation of VA interviewers to the deceased; place of interview; 4) time between death and VA interview; 5) description of the deceased (e.g. general population or suspected of a specific condition); 6) age composition of the deceased; 7) gender composition and other relevant characteristics of the deceased; 8) number of death records collected; 9) details of the respondent or source of the details of the deceased; 10) whether comparator was a primary or secondary data source; 11) description of the comparison data, 12) including number of records used; 13) categorization of quality of comparator dataset; 14) whether VA validation was the main study objective; 15) what measures were used in describing the agreement; 16) the findings of the agreement assessment; and 17) whether a computer coded or physician certified VA method was used.

The following categorisation was developed to assess the quality of the comparator dataset.

- Level 1: Autopsy diagnosis.
- Level 2: Hospital diagnosis with evidence suggestive that investigation findings (except autopsy) were used in the diagnosis, in addition to the signs and symptoms.
- Level 3: Hospital diagnosis without further details on the criteria used/ Hospital diagnosis only based on signs or symptoms.
- Level 4: Externally made diagnosis (e.g. by a physician not involved in the management of the patient) using available clinical records.
- Level 5: Civil registry or other non-medical records.

The identified domains were summarised using narrative synthesis under the domains; objective and settings, risk of bias, VA instruments and data collectors, study populations and analysis techniques including comparator dataset.

## Results

The characteristics of the selected studies are shown in Additional File 2.

### Objective and settings

Among the 66 studies, 59 included VA validation as the primary objective, whereas in 7 studies it was either a secondary objective, the comparator, or done on a sub-sample [14–20]. 10 validation studies were conducted on existing datasets [8, 9, 15, 21–27]. The other studies involved a data collection component in diverse countries including Ethiopia, South Africa, India, China, Philippines, Pakistan, Bangladesh, Georgia, Haiti, Ghana, Tanzania, Indonesia, Iran, Kenya, Zimbabwe, Liberia, Malawi, Malaysia, Mexico, Uganda and Vietnam.

### Risk of bias

The risk of bias assessment is summarized in Additional File 3. Most studies were low risk across the individual domains assessed, however, 9 studies [15, 28–35] did not collect a representative sample or cover the entire population (Domain 2), 17 studies [15, 17, 29, 30, 32, 35–46] had a high risk of non-response (Domain 3), and 15 studies [16, 17, 20, 31, 32, 40, 43, 46–53] did not use a validated instrument (Domain 7).

### VA instruments and data collectors

Except for the 10 studies [8, 9, 15, 21–27] that used existing databases, the most used VA instruments were the World Health Organization questionnaire (*n* = 16) [29, 39, 41, 51, 54–63] and Population Health Metrics Research Consortium (PHMRC) questionnaire (*n* = 5) [8, 42, 64–66]. In 7 studies [18, 40, 43, 48, 53, 67, 68], the tool was not mentioned. In other studies, locally designed VA instruments were used. In 5 studies [30, 32, 49, 50, 67], the data collectors were nurses, in another 3 [20, 51, 59] they were doctors, in 10 [14, 29, 38, 43, 60, 61, 64, 66, 69, 70] the type of data collectors was not mentioned. Other studies included several other categories such as field workers and non-medical graduates.

In the majority (*n* = 38) of validation studies, the data collection was conducted at the household setting of the deceased. In 4 studies [30, 35, 58, 71], a combination of settings was used. In 10 studies [28, 29, 40, 44, 45, 52, 64, 68–70], the data collection setting was not mentioned. Other data collection settings included during pilgrimage, in hospital, and at the mortuary. The time between death and the interview varied from immediately on collection of the body up to 42 months. In 20 studies [16–19, 28, 34, 35, 42, 45, 46, 48, 52, 53, 57, 59, 64, 67–70], the time between the death and the interview was not mentioned. The interviewees were not specified in 18 studies [20, 28, 29, 40, 43–46, 56, 58, 62, 64, 65, 67–71], however in all others they were clearly described as family members, relatives or principal caregivers at the time of death.

### Study populations

In 29 studies, the study population included the general adult population and in 15 studies [14, 30, 31, 33, 38, 39, 52, 54–56, 60, 61, 63, 70, 72] it included general neonatal or child deaths. Three studies included only female deaths [34, 47, 73] and others were conducted on selected groups such as deaths from HIV/TB, pilgrims, and respiratory infections. In general, the studies with general adult deaths had an approximately equal male to female ratio. In three studies there were fewer than 50 interviews [30, 53, 74], and in the others it ranged from 100 to over 26,000.

### Analysis techniques and comparator dataset

Physician certification was used in 36 studies and computer coded methods were used in 14 studies [8, 15, 18, 23, 24, 27, 29, 32, 42, 44, 50, 64, 66, 75]. Both techniques were used in 11 studies [21, 22, 28, 40, 47, 52, 58, 65, 70, 71, 76] and the method was not mentioned clearly in 6 studies [9, 26, 38, 45, 67, 68]. The comparator data was a primary source in the majority of studies (*n* = 53), while in 13 it was a secondary source.

Categorisation of quality of the comparator dataset is shown in Table 1. Most studies (*n* = 37) used a comparator dataset of high quality (level 1 or 2) that included investigation findings.

**Table 1:** Categorization of the quality of the comparator dataset

Level	Description	Frequency (%)	References
[1]	Autopsy diagnosis	1 (1.5%)	[53]
2	Hospital diagnosis with evidence suggestive that investigation findings (i.e. except autopsy) were used in the diagnosis in addition to the signs and symptoms	36 (54.5%)	[2–37]
3	Hospital diagnosis without further details of the criteria used/Hospital diagnosis only based on signs and symptoms	7 (10.6%)	[38–44]
4	Externally made diagnosis (e.g. by a physician not involved in the management of the patient) using available clinical records	11 (16.7%)	[45–55]
5	Civil registry or other non-medical records	8 (12.1%)	[56–63]
Mixed	More than 1 level	3 (4.5%)	[64–66]
Total		66 (100.0%)	

The measures used in the comparison included percentage agreement, chance-corrected concordance (CCC), CSMF accuracy, Kappa coefficient, concordance correlation coefficient, sensitivity, specificity, predictive value and statistical tests for exploring significant differences. Eleven studies [8, 21, 23–25, 27, 58, 64, 65, 71, 75] used CCC, 14 studies [8, 21–27, 58, 64, 65, 68, 71, 75] used CSMF accuracy, 16 studies [8, 18–20, 23, 28, 34, 39, 55, 57–60, 66, 67, 73] used kappa coefficient, and 38 studies [9, 16, 17, 20, 28, 29, 31–35, 37, 39, 40, 43–46, 48–50, 52–57, 59, 60, 62, 63, 66, 67, 69, 72, 73, 75, 77] used sensitivity, specificity, or positive predictive value. In general, the studies showed overall acceptable agreements. Specificity was observed to be higher than sensitivity in most of the studies that measured those two parameters. Additionally, the negative predictive value was higher than the positive predictive value.

## Discussion

This is the first systematic review conducted on the characteristics of VA validation studies. This review revealed that while most studies have confirmed the validity of VA methods, the quality of the comparator datasets varied considerably, impacting the interpretation of VA methods. Additionally, most studies used VA certified by physicians and a variety of VA instruments and agreement metrics.

This review highlights several factors related to the quality of VA validation methods. First, even though some studies were designated “validation studies”, on closer inspection, key criteria were missing. For example, in 7 studies, the main objective was not to validate the VA method. Further, in 13 studies the validation was conducted using a secondary data collection method in which the comparator did not include the same set of deaths. Essential details that should have been mentioned were missing for some studies. For example, the type of data collectors was not mentioned in 8 studies; the data collection setting was not mentioned in 10 studies; the time between death and interview was not mentioned in 20 studies; and the interviewees were not specified in 18 studies.

The second implication of this review is that the quality of the comparator data used needs to be evaluated carefully. We developed a new classification to assess the quality of comparator data assessment. Twenty-six studies used a lower quality comparator than level 2, meaning the comparator dataset did not include laboratory or imaging findings. For some causes of death, such as injuries or external causes of death, further investigations beyond the clinical record may not be necessary to determine cause of death. But for other causes, such as specific cancers, laboratory findings are essential to accurately assigning cause of death. The availability of laboratory and imaging investigations can be limited, especially in rural areas where VA is most often used, which presents a challenge for VA validation studies. Furthermore, most studies were not focused on external causes of death, which suggests higher quality comparison cause of death data is needed to reliably interpret the validity of VA methods.

Third, VA validation studies should adhere to more consistent use of agreement metrics. Most studies used sensitivity, specificity, or positive predictive value to evaluate the agreement for cause of death assignment. While these metrics are appropriate for some comparisons, they do not adjust for assignment by chance as in CCC and chance-corrected cause-specific mortality fraction [78, 79]. Additionally, since VA is a blunt tool, its primary purpose remains at population, rather than individual level cause of death assignment [80]. As such, validation studies should include a metric that assesses the population level agreement, such as CSMF accuracy.

There were several limitations of this review. Firstly, only articles in English were included in the review. In the risk of bias assessment, although the aim was to conduct objective assessments, the possibility of some subjectivity could not be excluded. As an example, one criterion was whether a validated tool was used. In reporting the studies, the exact phrase “validated tool” was used in some studies whereas in others, less specific descriptions were used, such as “modifications which were made to validated tools”.

## Conclusions

This review highlights that while the majority of VA validation studies have reported favourable findings for the VA method under assessment, major differences were observed in the methodologies. Many used poor quality comparison cause of death data that affects the interpretation of the validation assessment. Future VA validation studies should adhere to consistent methodological criteria. Robust validation studies will help health policy planners and those involved in VA implementation make informed decisions before selecting a particular VA method.

## Supplementary Information

**Additional file 1.** Search Strategies used in MEDLINE and EMBASE.

**Additional file 2.** Characteristics of all studies selected from the literature search.

**Additional file 3.** Colour coded risk of bias assessment across ten domains.

**Additional file 4. **

## Data Availability

All data generated or analysed during this study are included in this published article and its supplementary information files.

## Abbreviations

CSMF: Cause-specific mortality fraction
CCC: Chance-corrected concordance
PHMRC: Population Health Metrics Research Consortium
VA: Verbal autopsy
