# Correction: Validation studies of verbal autopsy methods: a systematic review

**DOI:** 10.1186/s12889-022-14895-y

**Published:** 2023-01-23

**Authors:** Buddhika P. K. Mahesh, John D. Hart, Ajay Acharya, Hafizur Rahman Chowdhury, Rohina Joshi, Tim Adair, Riley H. Hazard

**Affiliations:** 1 Melbourne School of Population and Global Health, The University of Melbourne, Carlton, Victoria Australia; 2 The George Institute for Global Health, New Delhi, India; 3 School of Population Health, University of New South Wales, Sydney, Australia

**Correction: BMC Public Health 22, 2215 (2022)**

**https://doi.org/10.1186/s12889-022-14628-1**

During the publication [1] process errors were introduced in the citations in table 1. These errors have been amended and the correct table is available in the original paper.
