# Supplementary material for: Validation studies of verbal autopsy methods: a systematic review
Source: BMC Public Health. 2022 Nov 29;22:2215. doi: 10.1186/s12889-022-14628-1 (PMC9706899; doi:10.1186/s12889-022-14628-1)
Supplement: Supplementary file 1 — Additional file 1. Search Strategies used in MEDLINE and EMBASE. [file 12889_2022_14628_MOESM1_ESM.docx]

**Search Strategies used in MEDLINE and EMBASE**

Database: Ovid MEDLINE(R) and Epub Ahead of Print, In-Process & Other Non-Indexed Citations and Daily <1946 to June 09, 2020>

Search Strategy:

--------------------------------------------------------------------------------

1 verbal autop*.mp. [mp=title, abstract, original title, name of substance word, subject heading word, floating sub-heading word, keyword heading word, organism supplementary concept word, protocol supplementary concept word, rare disease supplementary concept word, unique identifier, synonyms] (1015)

2 Validation Study/ or "Reproducibility of Results"/ (455967)

3 (validat* or comparat* or accuracy or agreement or concordance).mp. [mp=title, abstract, original title, name of substance word, subject heading word, floating sub-heading word, keyword heading word, organism supplementary concept word, protocol supplementary concept word, rare disease supplementary concept word, unique identifier, synonyms] (3131911)

4 2 or 3 (3330579)

5 1 and 4 (294)

***************************

Database: Embase Classic+Embase <1947 to 2020 Week 23>

Search Strategy:

--------------------------------------------------------------------------------

1 verbal autops*.mp. [mp=title, abstract, heading word, drug trade name, original title, device manufacturer, drug manufacturer, device trade name, keyword, floating subheading word, candidate term word] (1220)

2 cross validation/ or validation study/ or instrument validation/ or validation process/ (226591)

3 (validat* or comparat* or accuracy or agreement or concordance).mp. [mp=title, abstract, heading word, drug trade name, original title, device manufacturer, drug manufacturer, device trade name, keyword, floating subheading word, candidate term word] (3112026)

4 2 or 3 (3112026)

5 1 and 4 (307)

***************************
