# Supplementary material for: Validation studies of verbal autopsy methods: a systematic review
Source: BMC Public Health. 2022 Nov 29;22:2215. doi: 10.1186/s12889-022-14628-1 (PMC9706899; doi:10.1186/s12889-022-14628-1)
Supplement: Supplementary file 3 — Additional file 3. Colour coded risk of bias assessment across ten domains. [file 12889_2022_14628_MOESM3_ESM.docx]

**Additional File 3: Colour coded risk of bias assessment across ten domains**

Red - high risk, Yellow - unclear, Green - low risk

|  | Domains* | | | | | | | | | |
| --- | --- | --- | --- | --- | --- | --- | --- | --- | --- | --- |
| Study ID | 1 | 2 | 3 | 4 | 5 | 6 | 7 | 8 | 9 | 10 |
| Aggrawal et.al, 2011(1) |  |  |  |  |  |  |  |  |  |  |
| Aggarwal et al, 2013(2) |  |  |  |  |  |  |  |  |  |  |
| Arudo et al, 1998(3) |  |  |  |  |  |  |  |  |  |  |
| Bauni et al, 2011(4) |  |  |  |  |  |  |  |  |  |  |
| Byass, 2014(5) |  |  |  |  |  |  |  |  |  |  |
| Byass, 2013(6) |  |  |  |  |  |  |  |  |  |  |
| Chandramohan et al, 1998(A)(7) |  |  |  |  |  |  |  |  |  |  |
| Chandramohan et.al, 1998(B)(8) |  |  |  |  |  |  |  |  |  |  |
| Chowdhury et.al, 2019(9) |  |  |  |  |  |  |  |  |  |  |
| Coldham et al, 2000(10) |  |  |  |  |  |  |  |  |  |  |
| Dehghan et al, 2018(11) |  |  |  |  |  |  |  |  |  |  |
| Dowell et al, 1993 (12) |  |  |  |  |  |  |  |  |  |  |
| Edmond et.al, 2008(13) |  |  |  |  |  |  |  |  |  |  |
| Flaxman et al, 2011(A)(14) |  |  |  |  |  |  |  |  |  |  |
| Flaxman et.al ,2011(B)(15) |  |  |  |  |  |  |  |  |  |  |
| Gajalakshmi et.al, 2004(16) |  |  |  |  |  |  |  |  |  |  |
| Gajalakshmil et.al, 2002 (17) |  |  |  |  |  |  |  |  |  |  |
| Ganapathy et.al, 2017(18) |  |  |  |  |  |  |  |  |  |  |
| Hong et.al, 2018(19) |  |  |  |  |  |  |  |  |  |  |
| Hussain et.al, 2015(20) |  |  |  |  |  |  |  |  |  |  |
| James et.al, 2011(21) |  |  |  |  |  |  |  |  |  |  |
| Joubert et.al, 2014(22) |  |  |  |  |  |  |  |  |  |  |
| Kahn et.al, 2000(23) |  |  |  |  |  |  |  |  |  |  |
| Kalter et.al, 2016 (24) |  |  |  |  |  |  |  |  |  |  |
| Kalter et.al, 1990 (25) |  |  |  |  |  |  |  |  |  |  |
| Kamali et al, 1996(26) |  |  |  |  |  |  |  |  |  |  |
| Karat et al, 2018(27) |  |  |  |  |  |  |  |  |  |  |
| Karat et al, 2017 (28) |  |  |  |  |  |  |  |  |  |  |
| Khademi et al, 2010 (29) |  |  |  |  |  |  |  |  |  |  |
| Lomia et al, 2014(30) |  |  |  |  |  |  |  |  |  |  |
| Lopman et al, 2010(31) |  |  |  |  |  |  |  |  |  |  |
| Lopman et al, 2006(32) |  |  |  |  |  |  |  |  |  |  |
| Lozano et al, 2011(A)(33) |  |  |  |  |  |  |  |  |  |  |
| Lozano, et al 2011(B)(34) |  |  |  |  |  |  |  |  |  |  |
| Marsh et al, 2003(35) |  |  |  |  |  |  |  |  |  |  |
| Mayanja et al, 2011(36) |  |  |  |  |  |  |  |  |  |  |
| Midhet, 2008(37) |  |  |  |  |  |  |  |  |  |  |
| Misganaw 2012(38) |  |  |  |  |  |  |  |  |  |  |
| Mobley et al, 1996(39) |  |  |  |  |  |  |  |  |  |  |
| Mpimbaza et al 2011(40) |  |  |  |  |  |  |  |  |  |  |
| Mpimbaza et al 2015(41) |  |  |  |  |  |  |  |  |  |  |
| Murray et al 2011(42) |  |  |  |  |  |  |  |  |  |  |
| Murray et al 2007(43) |  |  |  |  |  |  |  |  |  |  |
| Murray et al 2014(44) |  |  |  |  |  |  |  |  |  |  |
| Murtaza et al 2019(45) |  |  |  |  |  |  |  |  |  |  |
| Nausheen et al 2013(46) |  |  |  |  |  |  |  |  |  |  |
| Ndila et al 2014(47) |  |  |  |  |  |  |  |  |  |  |
| Pacque-Margolis 1990(48) |  |  |  |  |  |  |  |  |  |  |
| Pane et al 2013(49) |  |  |  |  |  |  |  |  |  |  |
| Polprasert et al 2010(50) |  |  |  |  |  |  |  |  |  |  |
| Quigley et al 1996(51) |  |  |  |  |  |  |  |  |  |  |
| Quigley et al 1999(52) |  |  |  |  |  |  |  |  |  |  |
| Qureshi et al 2014(53) |  |  |  |  |  |  |  |  |  |  |
| Ramirez-Villalobos et al 2019(54) |  |  |  |  |  |  |  |  |  |  |
| Rodriguez et al 1998(55) |  |  |  |  |  |  |  |  |  |  |
| Serina et al 2015(56) |  |  |  |  |  |  |  |  |  |  |
| Serina et al 2015(57) |  |  |  |  |  |  |  |  |  |  |
| Setel et al 2006(58) |  |  |  |  |  |  |  |  |  |  |
| Soofi et al 2015(59) |  |  |  |  |  |  |  |  |  |  |
| Tensou et al 2010(60) |  |  |  |  |  |  |  |  |  |  |
| Tran et al 2018(61) |  |  |  |  |  |  |  |  |  |  |
| Wang et al 2007(62) |  |  |  |  |  |  |  |  |  |  |
| Yang et al 2006(63) |  |  |  |  |  |  |  |  |  |  |
| Yokobori et al 2020(64) |  |  |  |  |  |  |  |  |  |  |
| Akgun, Colak and Bakar 2012(65) |  |  |  |  |  |  |  |  |  |  |
| Mondal et al 2015(66) |  |  |  |  |  |  |  |  |  |  |

*Description of the domains:

Domain 1: Study’s target population/dataset involved was a close representation of the population of a defined geographical area/s (national or sub-national) OR a close representation of the population presenting to a healthcare institution/s in relation to socio-demographic characteristics

Domain 2: Random selection was used to select the sample OR the total target population/dataset was covered

Domain 3: Non-response bias was minimal

Domain 4: Appropriate descriptive statistics have been used in describing the comparison

Domain 5: Appropriate summary statistics have been used for the comparison

Domain 6: Comparator had been clearly described

Domain 7: A validated instrument (e.g. questionnaire) was used for data collection

Domain 8: Observers were blinded to previous findings

Domain 9: Comparator had been determined before the data collection

Domain 10: Clear descriptions have been given on the competence of those who were involved in data collection and analysis

**References**

1. Aggarwal AK, Jain V, Kumar R. Validity of verbal autopsy for ascertaining the causes of stillbirth. Bull World Health Organ. 2011;89(1):31-40.

2. Aggarwal AK, Kumar P, Pandit S, Kumar R. Accuracy of WHO verbal autopsy tool in determining major causes of neonatal deaths in India. PLoS One. 2013;8(1):e54865.

3. Arudo J, Gimnig JE, ter Kuile FO, Kachur SP, Slutsker L, Kolczak MS, et al. Comparison of government statistics and demographic surveillance to monitor mortality in children less than five years old in rural western Kenya. Am J Trop Med Hyg. 2003;68(4 Suppl):30-7.

4. Bauni E, Ndila C, Mochamah G, Nyutu G, Matata L, Ondieki C, et al. Validating physician-certified verbal autopsy and probabilistic modeling (InterVA) approaches to verbal autopsy interpretation using hospital causes of adult deaths. Popul Health Metr. 2011;9:49.

5. Byass P. Usefulness of the Population Health Metrics Research Consortium gold standard verbal autopsy data for general verbal autopsy methods. BMC Med. 2014;12:23.

6. Byass P, Calvert C, Miiro-Nakiyingi J, Lutalo T, Michael D, Crampin A, et al. InterVA-4 as a public health tool for measuring HIV/AIDS mortality: a validation study from five African countries. Glob Health Action. 2013;6:22448.

7. Chandramohan D, Maude GH, Rodrigues LC, Hayes RJ. Verbal autopsies for adult deaths: their development and validation in a multicentre study. Trop Med Int Health. 1998;3(6):436-46.

8. Chandramohan D, Rodrigues LC, Maude GH, Hayes RJ. The validity of verbal autopsies for assessing the causes of institutional maternal death. Stud Fam Plann. 1998;29(4):414-22.

9. Chowdhury HR, Flaxman AD, Joseph JC, Hazard RH, Alam N, Riley ID, et al. Robustness of the Tariff method for diagnosing verbal autopsies: impact of additional site data on the relationship between symptom and cause. BMC Med Res Methodol. 2019;19(1):232.

10. Coldham C, Ross D, Quigley M, Segura Z, Chandramohan D. Prospective validation of a standardized questionnaire for estimating childhood mortality and morbidity due to pneumonia and diarrhoea. Trop Med Int Health. 2000;5(2):134-44.

11. Dehghan A, Nasirian M, Haghdoost AA, Bahramali E, Sharifi H. Validation of the verbal autopsy questionnaire for adult deaths in Iran. Med J Islam Repub Iran. 2018;32:7.

12. Dowell SF, Davis HL, Holt EA, Ruff AJ, Kissinger PJ, Bijoux J, et al. The utility of verbal autopsies for identifying HIV-1-related deaths in Haitian children. AIDS. 1993;7(9):1255-9.

13. Edmond KM, Quigley MA, Zandoh C, Danso S, Hurt C, Owusu Agyei S, et al. Diagnostic accuracy of verbal autopsies in ascertaining the causes of stillbirths and neonatal deaths in rural Ghana. Paediatr Perinat Epidemiol. 2008;22(5):417-29.

14. Flaxman AD, Vahdatpour A, Green S, James SL, Murray CJ, Population Health Metrics Research C. Random forests for verbal autopsy analysis: multisite validation study using clinical diagnostic gold standards. Popul Health Metr. 2011;9:29.

15. Flaxman AD, Vahdatpour A, James SL, Birnbaum JK, Murray CJ, Population Health Metrics Research C. Direct estimation of cause-specific mortality fractions from verbal autopsies: multisite validation study using clinical diagnostic gold standards. Popul Health Metr. 2011;9:35.

16. Gajalakshmi V, Peto R. Verbal autopsy of 80,000 adult deaths in Tamilnadu, South India. BMC Public Health. 2004;4:47.

17. Gajalakshmi V, Peto R, Kanaka S, Balasubramanian S. Verbal autopsy of 48 000 adult deaths attributable to medical causes in Chennai (formerly Madras), India. BMC Public Health. 2002;2:7.

18. Ganapathy SS, Yi Yi K, Omar MA, Anuar MFM, Jeevananthan C, Rao C. Validation of verbal autopsy: determination of cause of deaths in Malaysia 2013. BMC Public Health. 2017;17(1):653.

19. Hong TT, Phuong Hoa N, Walker SM, Hill PS, Rao C. Completeness and reliability of mortality data in Viet Nam: Implications for the national routine health management information system. PLoS One. 2018;13(1):e0190755.

20. Hussain-Alkhateeb L, Fottrell E, Petzold M, Kahn K, Byass P. Local perceptions of causes of death in rural South Africa: a comparison of perceived and verbal autopsy causes of death. Glob Health Action. 2015;8:28302.

21. James SL, Flaxman AD, Murray CJ, Population Health Metrics Research C. Performance of the Tariff Method: validation of a simple additive algorithm for analysis of verbal autopsies. Popul Health Metr. 2011;9:31.

22. Joubert J, Bradshaw D, Kabudula C, Rao C, Kahn K, Mee P, et al. Record-linkage comparison of verbal autopsy and routine civil registration death certification in rural north-east South Africa: 2006-09. Int J Epidemiol. 2014;43(6):1945-58.

23. Kahn K, Tollman SM, Garenne M, Gear JS. Validation and application of verbal autopsies in a rural area of South Africa. Trop Med Int Health. 2000;5(11):824-31.

24. Kalter HD, Perin J, Black RE. Validating hierarchical verbal autopsy expert algorithms in a large data set with known causes of death. J Glob Health. 2016;6(1):010601.

25. Kalter HD, Gray RH, Black RE, Gultiano SA. Validation of postmortem interviews to ascertain selected causes of death in children. Int J Epidemiol. 1990;19(2):380-6.

26. Kamali A, Wagner HU, Nakiyingi J, Sabiiti I, Kengeya-Kayondo JF, Mulder DW. Verbal autopsy as a tool for diagnosing HIV-related adult deaths in rural Uganda. Int J Epidemiol. 1996;25(3):679-84.

27. Karat AS, Maraba N, Tlali M, Charalambous S, Chihota VN, Churchyard GJ, et al. Performance of verbal autopsy methods in estimating HIV-associated mortality among adults in South Africa. BMJ Glob Health. 2018;3(4):e000833.

28. Karat AS, Tlali M, Fielding KL, Charalambous S, Chihota VN, Churchyard GJ, et al. Measuring mortality due to HIV-associated tuberculosis among adults in South Africa: Comparing verbal autopsy, minimally-invasive autopsy, and research data. PLoS One. 2017;12(3):e0174097.

29. Khademi H, Etemadi A, Kamangar F, Nouraie M, Shakeri R, Abaie B, et al. Verbal autopsy: reliability and validity estimates for causes of death in the Golestan Cohort Study in Iran. PLoS One. 2010;5(6):e11183.

30. Lomia N, Berdzuli N, Sturua L, Kereselidze M, Topuridze M, Pestvenidze E, et al. Leading causes of death of women of reproductive age in the Republic of Georgia: findings from the National Reproductive Age Mortality Survey (2014). Int J Womens Health. 2018;10:437-52.

31. Lopman B, Cook A, Smith J, Chawira G, Urassa M, Kumogola Y, et al. Verbal autopsy can consistently measure AIDS mortality: a validation study in Tanzania and Zimbabwe. J Epidemiol Community Health. 2010;64(4):330-4.

32. Lopman BA, Barnabas RV, Boerma JT, Chawira G, Gaitskell K, Harrop T, et al. Creating and validating an algorithm to measure AIDS mortality in the adult population using verbal autopsy. PLoS Med. 2006;3(8):e312.

33. Lozano R, Freeman MK, James SL, Campbell B, Lopez AD, Flaxman AD, et al. Performance of InterVA for assigning causes of death to verbal autopsies: multisite validation study using clinical diagnostic gold standards. Popul Health Metr. 2011;9:50.

34. Lozano R, Lopez AD, Atkinson C, Naghavi M, Flaxman AD, Murray CJ, et al. Performance of physician-certified verbal autopsies: multisite validation study using clinical diagnostic gold standards. Popul Health Metr. 2011;9:32.

35. Marsh DR, Sadruddin S, Fikree FF, Krishnan C, Darmstadt GL. Validation of verbal autopsy to determine the cause of 137 neonatal deaths in Karachi, Pakistan. Paediatr Perinat Epidemiol. 2003;17(2):132-42.

36. Mayanja BN, Baisley K, Nalweyiso N, Kibengo FM, Mugisha JO, Van der Paal L, et al. Using verbal autopsy to assess the prevalence of HIV infection among deaths in the ART period in rural Uganda: a prospective cohort study, 2006-2008. Popul Health Metr. 2011;9:36.

37. Midhet F. Validating the verbal autopsy questionnaire for maternal mortality in pakistan. Int J Health Sci (Qassim). 2008;2(1):91-6.

38. Misganaw A, Mariam DH, Araya T, Aneneh A. Validity of verbal autopsy method to determine causes of death among adults in the urban setting of Ethiopia. BMC Med Res Methodol. 2012;12:130.

39. Mobley CC, Boerma JT, Titus S, Lohrke B, Shangula K, Black RE. Validation study of a verbal autopsy method for causes of childhood mortality in Namibia. J Trop Pediatr. 1996;42(6):365-9.

40. Mpimbaza A, Filler S, Katureebe A, Kinara SO, Nzabandora E, Quick L, et al. Validity of verbal autopsy procedures for determining malaria deaths in different epidemiological settings in Uganda. PLoS One. 2011;6(10):e26892.

41. Mpimbaza A, Filler S, Katureebe A, Quick L, Chandramohan D, SG S. Verbal Autopsy: Evaluation of Methods to Certify Causes of Death in Uganda. PLoS ONE 2015;10( e0128801).

42. Murray CJ, James SL, Birnbaum JK, Freeman MK, Lozano R, Lopez AD, et al. Simplified Symptom Pattern Method for verbal autopsy analysis: multisite validation study using clinical diagnostic gold standards. Popul Health Metr. 2011;9:30.

43. Murray CJ, Lopez AD, Feehan DM, Peter ST, Yang G. Validation of the symptom pattern method for analyzing verbal autopsy data. PLoS Med. 2007;4(11):e327.

44. Murray CJ, Lozano R, Flaxman AD, Serina P, Phillips D, Stewart A, et al. Using verbal autopsy to measure causes of death: the comparative performance of existing methods. BMC Med. 2014;12:5.

45. Murtaza SS, Kolpak P, Bener A, Jha P. Automated verbal autopsy classification: using one-against-all ensemble method and Naive Bayes classifier. Gates Open Res. 2018;2:63.

46. Nausheen S, Soofi SB, Sadiq K, Habib A, Turab A, Memon Z, et al. Validation of verbal autopsy tool for ascertaining the causes of stillbirth. PLoS One. 2013;8(10):e76933.

47. Ndila C, Bauni E, Nyirongo V, Mochamah G, Makazi A, Kosgei P, et al. Verbal autopsy as a tool for identifying children dying of sickle cell disease: a validation study conducted in Kilifi district, Kenya. BMC Med. 2014;12:65.

48. Pacque-Margolis S, Pacque M, Dukuly Z, Boateng J, Taylor HR. Application of the verbal autopsy during a clinical trial. Soc Sci Med. 1990;31(5):585-91.

49. Pane M, Imari S, Alwi Q, Nyoman Kandun I, Cook AR, Samaan G. Causes of mortality for Indonesian Hajj Pilgrims: comparison between routine death certificate and verbal autopsy findings. PLoS One. 2013;8(8):e73243.

50. Polprasert W, Rao C, Adair T, Pattaraarchachai J, Porapakkham Y, Lopez AD. Cause-of-death ascertainment for deaths that occur outside hospitals in Thailand: application of verbal autopsy methods. Popul Health Metr. 2010;8:13.

51. Quigley MA, Armstrong Schellenberg JR, Snow RW. Algorithms for verbal autopsies: a validation study in Kenyan children. Bull World Health Organ. 1996;74(2):147-54.

52. Quigley MA, Chandramohan D, Rodrigues LC. Diagnostic accuracy of physician review, expert algorithms and data-derived algorithms in adult verbal autopsies. Int J Epidemiol. 1999;28(6):1081-7.

53. Qureshi JS, Samuel JC, Mulima G, Kakoulides S, Cairns B, Charles AG. Validating a verbal autopsy tool to assess pre-hospital trauma mortality burden in a resource-poor setting. Trop Med Int Health. 2014;19(4):407-12.

54. Ramirez-Villalobos D, Stewart AL, Romero M, Gomez S, Flaxman AD, Hernandez B. Analysis of causes of death using verbal autopsies and vital registration in Hidalgo, Mexico. PLoS One. 2019;14(7):e0218438.

55. Rodriguez L, Reyes H, Tome P, Ridaura C, Flores S, Guiscafre H. Validation of the verbal autopsy method to ascertain acute respiratory infection as cause of death. Indian J Pediatr. 1998;65(4):579-84.

56. Serina P, Riley I, Stewart A, Flaxman AD, Lozano R, Mooney MD, et al. A shortened verbal autopsy instrument for use in routine mortality surveillance systems. BMC Med. 2015;13:302.

57. Serina P, Riley I, Stewart A, James SL, Flaxman AD, Lozano R, et al. Improving performance of the Tariff Method for assigning causes of death to verbal autopsies. BMC Med. 2015;13:291.

58. Setel PW, Whiting DR, Hemed Y, Chandramohan D, Wolfson LJ, Alberti KG, et al. Validity of verbal autopsy procedures for determining cause of death in Tanzania. Trop Med Int Health. 2006;11(5):681-96.

59. Soofi SB, Ariff S, Khan U, Turab A, Khan GN, Habib A, et al. Diagnostic accuracy of WHO verbal autopsy tool for ascertaining causes of neonatal deaths in the urban setting of Pakistan: a hospital-based prospective study. BMC Pediatr. 2015;15:144.

60. Tensou B, Araya T, Telake DS, Byass P, Berhane Y, Kebebew T, et al. Evaluating the InterVA model for determining AIDS mortality from verbal autopsies in the adult population of Addis Ababa. Trop Med Int Health. 2010;15(5):547-53.

61. Tran HT, Nguyen HP, Walker SM, Hill PS, Rao C. Validation of verbal autopsy methods using hospital medical records: a case study in Vietnam. BMC Med Res Methodol. 2018;18(1):43.

62. Wang L, Yang G, Jiemin M, Rao C, Wan X, Dubrovsky G, et al. Evaluation of the quality of cause of death statistics in rural China using verbal autopsies. J Epidemiol Community Health. 2007;61(6):519-26.

63. Yang G, Rao C, Ma J, Wang L, Wan X, Dubrovsky G, et al. Validation of verbal autopsy procedures for adult deaths in China. Int J Epidemiol. 2006;35(3):741-8.

64. Yokobori Y, Matsuura J, Sugiura Y, Mutemba C, Nyahoda M, Mwango C, et al. Analysis of causes of death among brought-in-dead cases in a third-level Hospital in Lusaka, Republic of Zambia, using the tariff method 2.0 for verbal autopsy: a cross-sectional study. BMC Public Health. 2020;20(1):473.

65. Akgun S, Colak M, Bakar C. Identifying and verifying causes of death in Turkey: National verbal autopsy survey. Public Health. 2012;126(2):150-8.

66. Mondal RN, Alam S, Haque A, Azad SJAK, Rani MRM, Shaeheduzzaman BA, et al. Validity of Verbal Autopsy for Assessment of Causes of Death in Bangladesh. European Academic Research. 2015;3(8).
